# Magnetic resonance imaging of the knee anteromedial quadrant

**DOI:** 10.1186/s12891-023-06732-z

**Published:** 2023-07-20

**Authors:** Pedro Baches Jorge, Rafael Baches Jorge, Diego Escudeiro de Oliveira, Camilo Partezani Helito, Lucas Nakazone Matos da Silva, Fernanda Tami Sato, Deivis Silva Brito, Igor Possebom

**Affiliations:** 1 Irmandade da Santa Casa de Misericórdia de São Paulo, São Paulo, Brazil; 2 Hospital das Clínicas da Faculdade de Medicina da Universidade de São Paulo, São Paulo, Brazil

**Keywords:** Anterior oblique ligament, Anteromedial quadrant, Posteromedial corner, Anteromedial rotatory instability

## Abstract

**Objective:**

This study aims to evaluate the possibility of characterizing an extra-articular thickening in the knee anteromedial quadrant in routine MRI scans.

**Materials and methods:**

Firstly, in a pilot study, for a better understanding of this extra-articular thickening trajectory in MRI, polytetrafluoroethylene (PTFE) tubes were attached to the ligament structure topography in two dissected pieces. Afterward, 100 knee MRI studies were randomly selected from our database, and 97 met the inclusion criteria. Two musculoskeletal radiologists interpreted the exams separately. Both had previously studied the ligament in the cadaveric knee MRI with the PTFE tube.

**Results:**

The **intraobserver** and interobserver agreement for the ligament identification was calculated using Cohen’s Kappa coefficient. The first radiologist identified the structure in 41 of the 97 scans (42.2%), and the second radiologist in 38 scans (39.2%). The interobserver agreement was substantial, with a Kappa of 0.68 and an agreement of 84.5%. The results suggest that this extra-articular thickening, recently called Anterior Oblique Ligament (AOL) in the literature, is a structure that can be frequently visualized on MRI scans with a high level of interobserver agreement in a relatively large number of exams.

**Conclusion:**

Therefore, this study indicates that MRI is a promising method for evaluating this anteromedial thickening, and it may be used for future studies of the Anterior Oblique Ligament.

## Introduction

Much has been studied about the complex anatomy of the lateral aspect of the knee, the most recent discovery being the anterolateral ligament, thoroughly studied on Magnetic Resonance Imaging by Porrino et al. in 2015 [1] and Patel et al. in 2017 [2]. On the other hand, when it comes to the medial compartment, there is a well-established and comprehended anatomy of its posteromedial corner but not of its anteromedial quadrant.

The posteromedial corner, which includes the posterior oblique ligament (POL), meniscotibial ligaments, oblique popliteal ligament, posterior horn of the medial meniscus, and an arm of the semimembranosus [3], is known to be an essential restrictor of medial stability (AMRI) over the entire knee range of motion [4, 5].

Noticing the absence of supporting structures in the knee anteromedial quadrant in literature, limited by the medial border of the patellar ligament, anterior border of the superficial medial collateral ligament (sMCL), and distal border of the medial patellar retinaculum, Jorge et al. [3], who had already observed a ligament-like formation in previous knee dissections, suspected the existence of a structure in the region that could complete their Theory of the Tibial Quadrants, in which he described that rotational control of the knee is maintained by the complementary function of diagonally opposite quadrants [3]. His team meticulously explored the anteromedial compartment by dissecting several cadaveric knees specimen, and very recently, in June 2022, reported an extra-articular thickening, histologically likewise a ligamentous structure in that region, located superficial to the sMCL never described before in the literature, which may assist in the restriction of external rotation and valgus motion of the knee. The newly discovered structure was named the Anterior Oblique Ligament (AOL) [3].

Based on the anatomical description of the AOL by Jorge et al., and its correlation on MRI in joint work with the Diagnostic Imaging Department, the question raised was whether it was possible to characterize the newly described ligament in routine knee MRI scans.

Therefore, this study aims to analyze the presence of AOL in MRI scans of patients who have undergone routine exams for different reasons.

## Materials and methods

A total of 100 knee MRI studies from patients of the institution were randomly selected from our database retrospectively. The exams were performed between February 2020 and May 2020, representing 21% of the knee MRI scans performed during the same period, using a 1.5 Tesla MRI with a 6-channel dedicated knee coil, following the institution’s routine knee MRI scanning protocol, as detailed in Table 1.

**Table 1:** Knee MRI scanning protocol

	Field of view	Matrix	TE	TR	Number of excitations	Slice thickness	Intersection gap
Coronal T1	160 × 160	384 × 384	12	467	1	3,5 mm	0,7 mm (20% de 3,5)
Coronal T2 PD FAT SAT	160 × 160	256 × 256	33	2090	1	3,5 mm	0,7 mm
Sagittal PD	160 × 160	256 × 256	33	2500	1	3,5 mm	0,7 mm
Sagittal T2 PD FAT SAT	160 × 160	256 × 256	33	2500	1	3,5 mm	0,7 mm
Axial T2 PD FAT SAT	150 × 150	256 × 256	44	3730	1	3,5 mm	0,35 mm (10%)

**Fig. 1:**
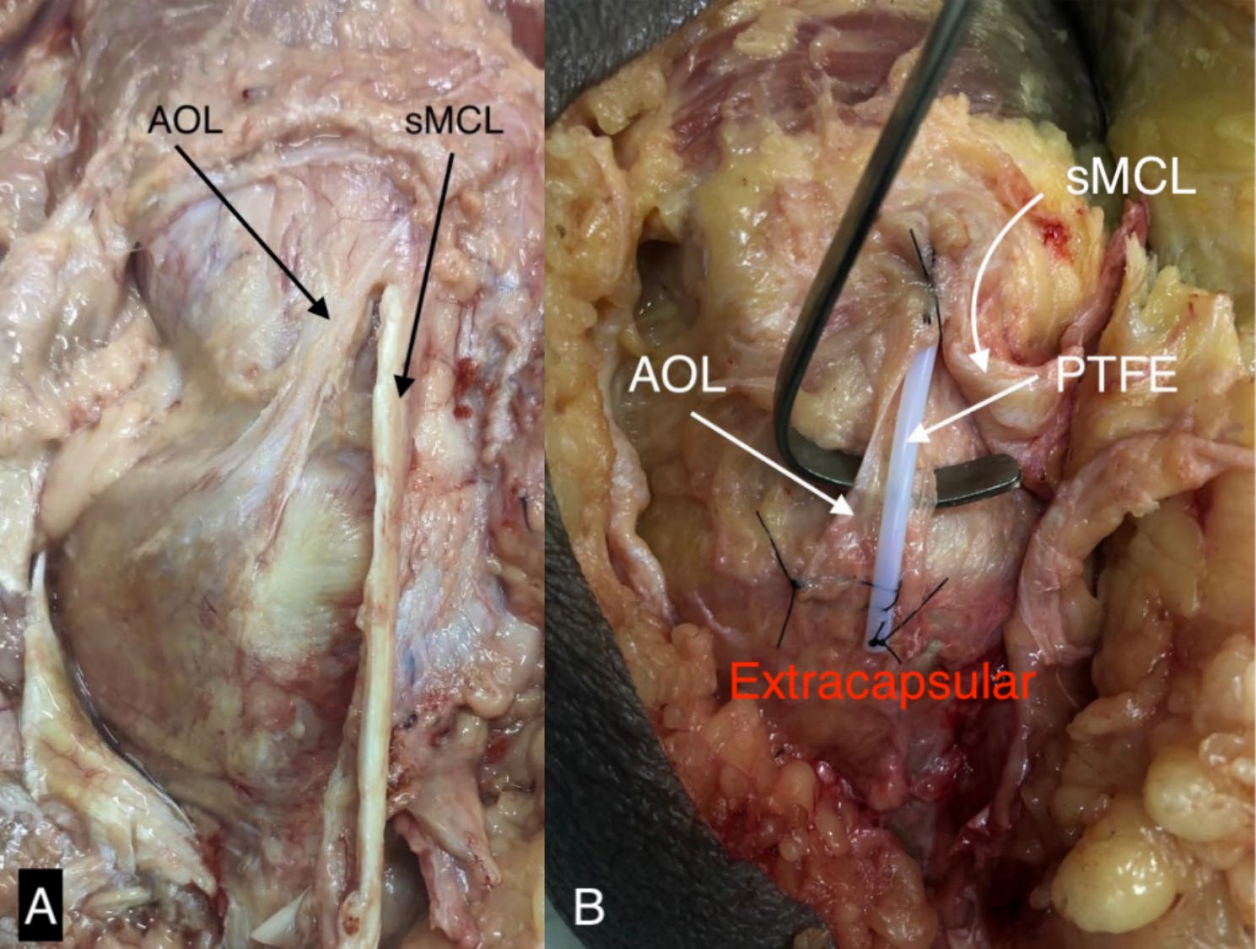
Cadaveric dissection of the knee medial aspect, showing the Anterior Oblique Ligament (AOL, Fig **A**) and Medial Collateral Ligament (MCL, Fig **A**). A polytetrafluoroethylene (PTFE, Fig **B**) tube was attached to the ligament for better visualization in MRI scans

Previously, for a better understanding of the AOL trajectory in MRI, a pilot study was carried out. Two cadaveric specimens of the entire knee derived from transfemoral amputations were submitted to MRI, using a 1.5 Tesla MRI with a 6-channel dedicated knee coil and evaluated in T1 and T2-weighted sequences, both in the axial, sagittal, and coronal planes. After acquiring the initial images, both pieces were dissected as described by Jorge et al., and a polytetrafluoroethylene (PTFE) tube was attached to the AOL (Fig. 1).

**Fig. 2:**
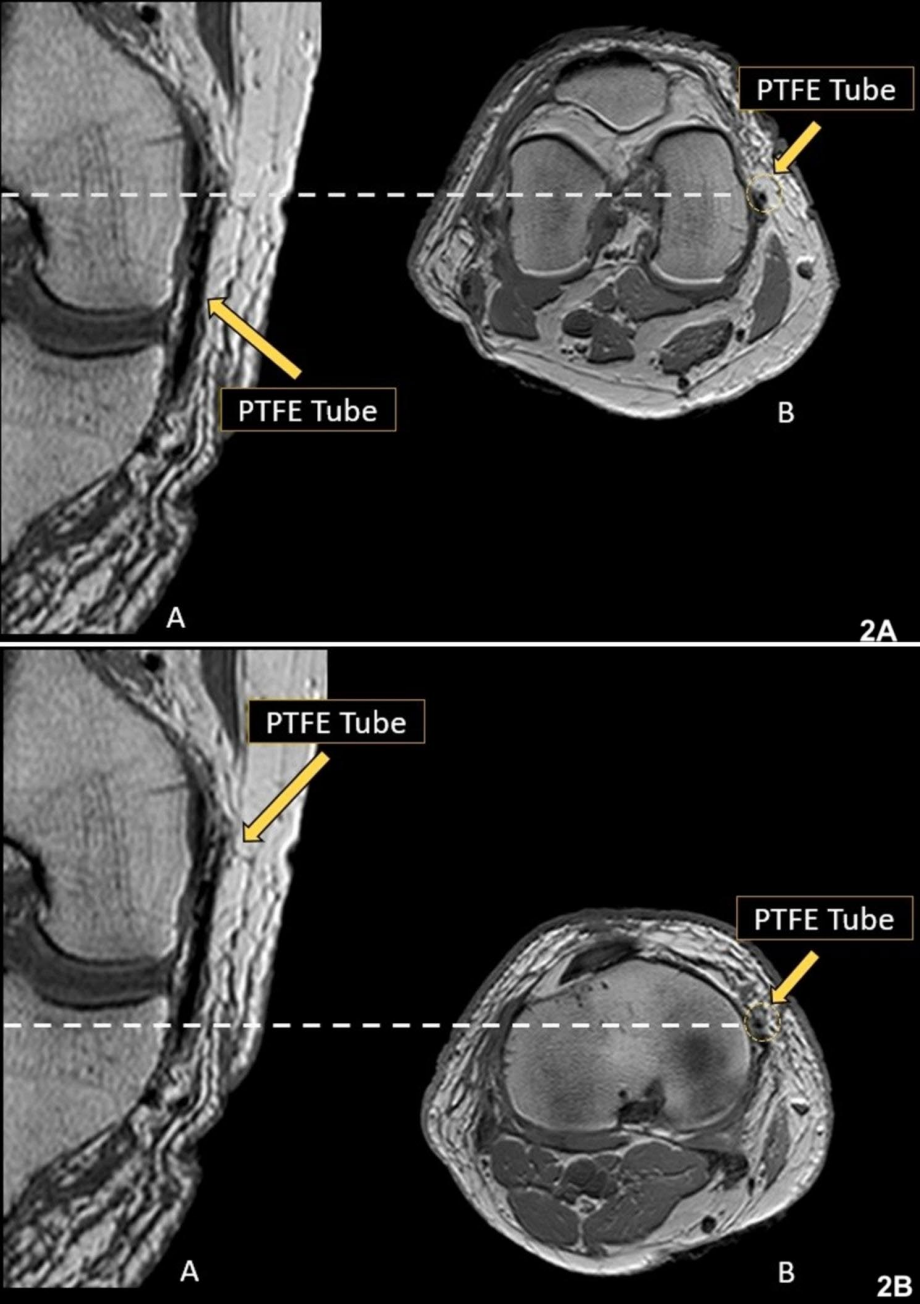
MRI scans with PTFE tube in the AOL proximal (**2A**) and distal (**2B**) topography

After the dissection, a new MRI scan was performed on the same day to study the path of the ligament with the PTFE tube, which made it more evident (Fig. 2a and b). The PTFE tube has a low signal intensity on T1 and T2-weighted sequences, enabling better visualization of the ligament path in MRI studies.

After that, two board-certified musculoskeletal radiologists, the first with ten years of experience (radiologist 1) and the second with seven years of experience (radiologist 2), studied together the trajectory of the ligament through the scans of knee specimens with the PTFE tube.

Exclusion criteria were the presence of medial collateral ligament (MCL) tear, expansile formations of the bone, soft tissue masses, and post-operative knees, which could alter the knee morphology or be associated with AOL tear. Studies considered normal and presenting other types of pathologies, such as degenerative and inflammatory/infectious diseases and trauma without MCL tears, were included in the analysis to avoid selection bias.

The 100 exams were interpreted separately by the two specialists, where each one evaluated the presence of the anterior oblique ligament and was allowed to visualize the different orthogonal planes simultaneously. No correlation with specific structures or pathologies was made at this study stage. The intraobserver and interobserver ligament identification agreement was then calculated using Cohen’s Kappa coefficient.

## Results

Three exams were excluded, two with MCL tear and one with ACL reconstruction. Therefore, a total of 97 studies were evaluated, 53 women and 44 men, with patient ages ranging from 13 to 68 years old (mean age of 35).

The first radiologist identified the AOL in 41 of the 97 MRI scans, with an incidence of 42.2%. 100% of the identified ligaments (41/41), were visualized in axial sequences, and 41.4% (17/41) in coronal sequences. (Table 2).

The second radiologist identified the AOL in 38 of the 97 MRI scans, with an incidence of 39.2%. The identified ligaments were also visualized in 100% (38/38) of the axial sequences and 31.6% (12/38) of the coronal sequences. (Table 2).

**Table 2:** Identification of AOL for both observers

	Radiologist 1	Radiologist 2
Presence AOL	41/97 (42,2%)	38/97 (39,2%)
Axial	41/41 (100%)	38/38 (100%)
coronal	17/41 (41,4%)	12/38 (31,6%)

The better visualization of the ligament in the axial sequence might be because of its orientation, however, the slice gap in the coronal scan protocol may have contributed to the obtained result.

**Fig. 3:**
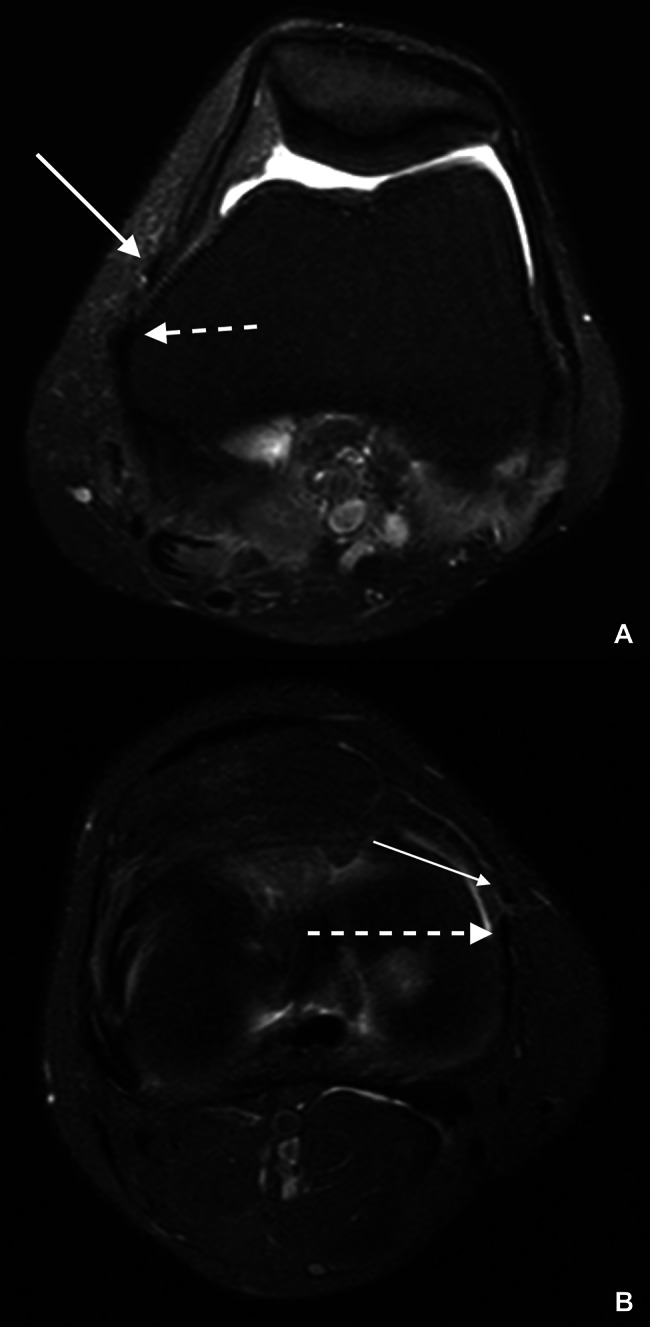
MRI axial scans showing the MCL (dashed arrow) and the AOL (closed arrow) at the proximal (**3A**) and distal (**3B**) topography

For both observers, most of the ligaments were better visualized in its middle third, whether it was on the axial or coronal plane. However, it can also be viewed in proximal and distal topography (Fig. 3a and b). The AOL femoral origin and tibial insertion were not always accurately identified.

### Intraobserver agreement

The interobserver agreement for identifying AOL on MRI studies was considered substantial (0.61–0.80), with a Kappa of 0.68 (95% confidence interval 0.532–0.828).

## Discussion

**Fig. 4:**
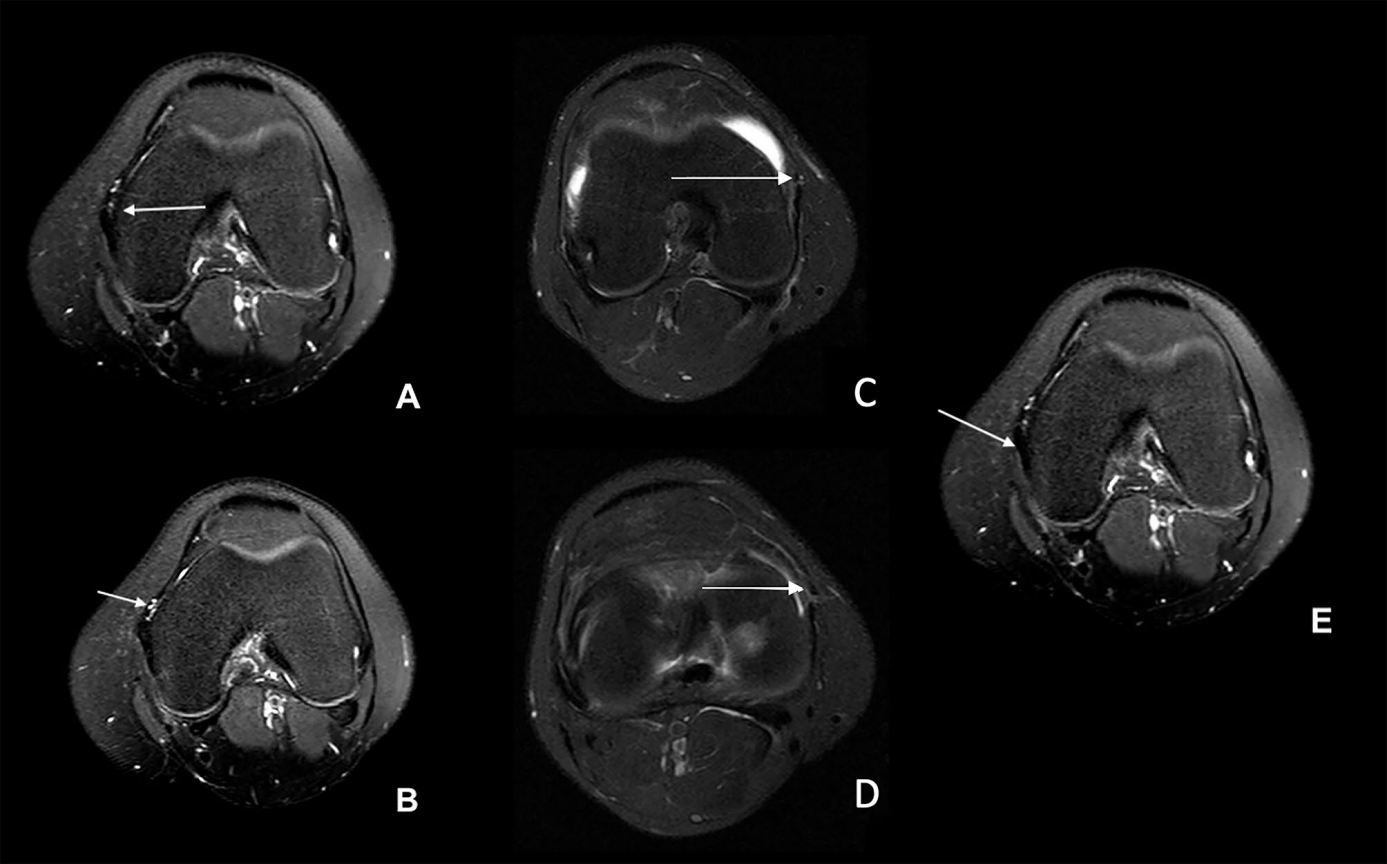
Tips for AOL identification (Table 3)

The main finding of this study is that the anterior oblique ligament (AOL) can be identified as a distinct structure in the anteromedial aspect of the knee in MRI exams. This structure was first observed by Jorge PB et al. [3] in a series of 21 knee dissections and shown to be histologically compatible with ligamentous tissue. Based on this series, Table 3; Fig. 4 provide tips for finding the Anterior Oblique Ligament.

**Table 3:** Tips for Anterior Oblique Ligament Identification on Axial Imaging (Fig. 4)

• Identify medial epicondyle - A
• Note the close relationship with the medial superior geniculate artery - B
• Note the anteroinferior orientation of AOL fibers - C and D
• Identify MCL anterior fibers - E

The medial side’s knee anatomy was first described by Warren and Marshal in layers, in a classic paper [6]. Recently, LaPrade et al. published a new anatomic description, based on their dissections [7]. The anteromedial quadrant was always described as an empty space, without ligamentous structures. It started to change when Peez C et described a structure in the anteromedial topography that would be the inferior extension of the medial patellar retinaculum [8]. At the same year (2022), Jorge et al. described in this region, the Anterior Oblique Ligament (AOL) [3]. The finding of this newly reported ligament, that lies anterior to the sMCL and has ligamentous histology, takes a step forward to understanding the knee rotational control, which they nominated the Theory of the Tibial Quadrants. According to that, the tibial plateau can be divided by two lines, anterior-posterior and medial-lateral, into four quadrants, with diagonally opposite quadrants acting in complementary functions. The reported ligament AOL and the anterior segment of the sMCL are located in the anterior medial quadrant and restrict valgus and external tibial rotation. Its opposite posterior lateral quadrant is composed of the popliteus tendon and popliteal-fibular ligament, restricting varus and external rotation. In the posterior medial compartment are included the posterior segment of the sMCL, posterior oblique ligament (POL), popliteal oblique ligament, and the semimembranosus. On its diagonal, the iliotibial tract and anterolateral ligament are the restrictors of varus and internal tibial rotation in the anterior lateral quadrant. Lastly, the lateral collateral ligament located on the lateral contour of the tibia controls the varus exclusively.

When we think about the ACL mechanism of trauma, in the majority of the cases occur in a flexed knee with a valgus and external rotation moment [9]. In these cases, a concomitant medial lesion is common [10], and due its rotational nature, goes from anterior to posterior. The Anteromedial rotational instability (AMRI) is known since1968, when Slocum and Larson published their paper [11], and the anterior sMCL portion play a role in it control [12]. The AOL, in the anteromedial quadrant, may be important in controlling the external rotation and valgus, and its reconstruction could be helpful in patients with ACL lesions and AMRI.

While biomechanical studies to evaluate the functional significance of the AOL are still in progress, the ability to visualize the ligament in non-invasive exams, essentially MRI, is also of extreme importance so that its analysis can be reproduced in the daily practice of knee assessment. With that said, our study aimed to investigate whether it was possible to visualize the ligament on MRI images of the knee and its incidence in the exams.

**Fig. 5:**
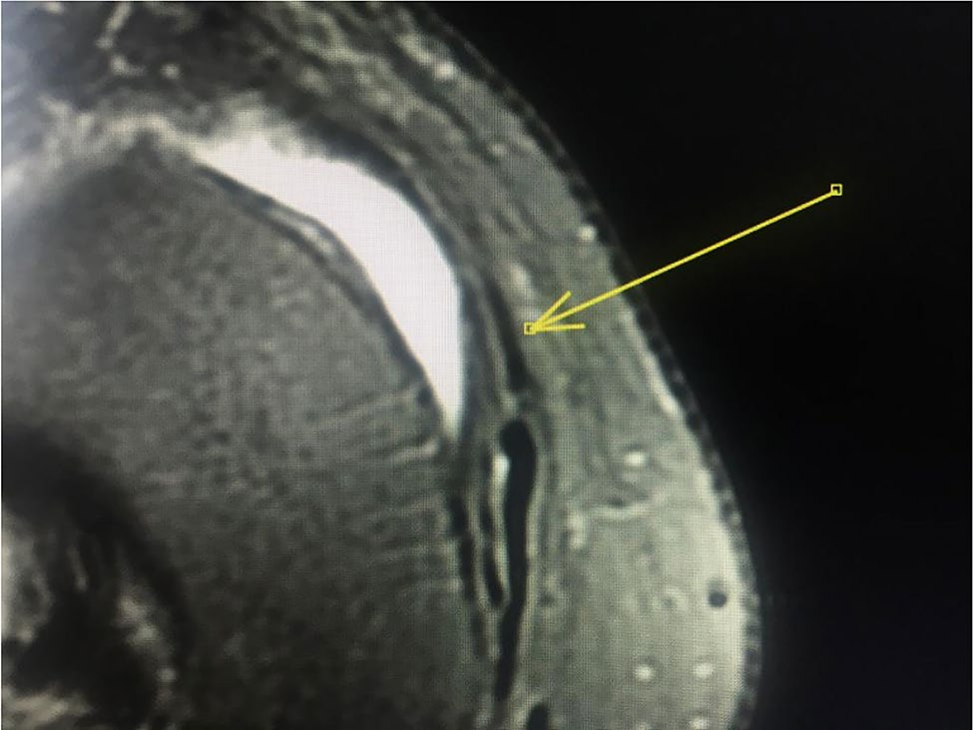
Axial MRI scan showing the AOL(arrow), anterior to the deep and superficial MCL

**Fig. 6:**
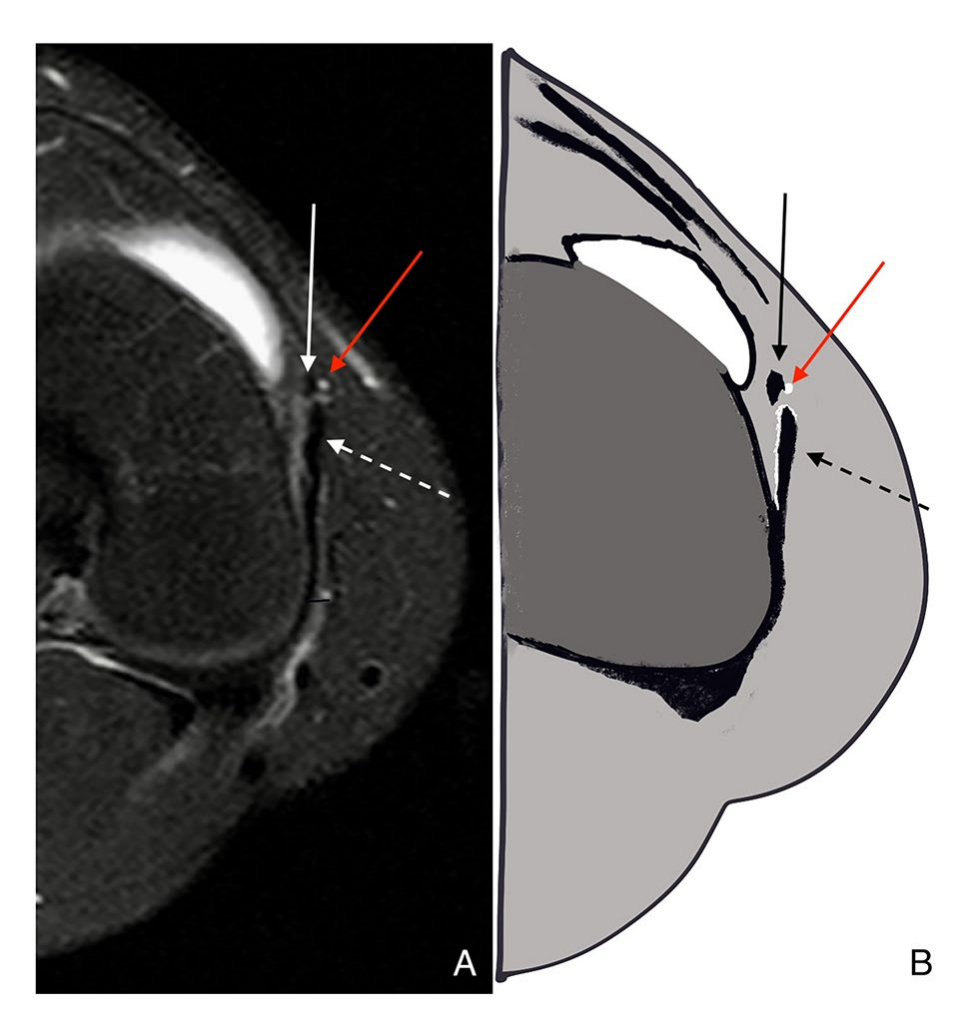
(**A**) coronal MRI slice; (**B**) Schematic drawing. Representing the AOL (closed arrow), the MCL (dashed arrow), and the close relationship with the medial superior geniculate artery (red arrow)

After the analysis of the MRI scans by the two radiologists, it was reached an agreement that the best method to visualize the ligament on axial images is to find a structure originating in the anterior aspect of the medial epicondyle of the femur, close to the medial superior geniculate artery, with fibers next to the anterior border of the superficial medial collateral ligament (sMCL) posteriorly Figs. 5 and 6. Dividing the medial side in 3 layers, like De Maeseneer et al. [13], the AOL is in the second one, anterior to the sMCL.

**Fig. 7:**
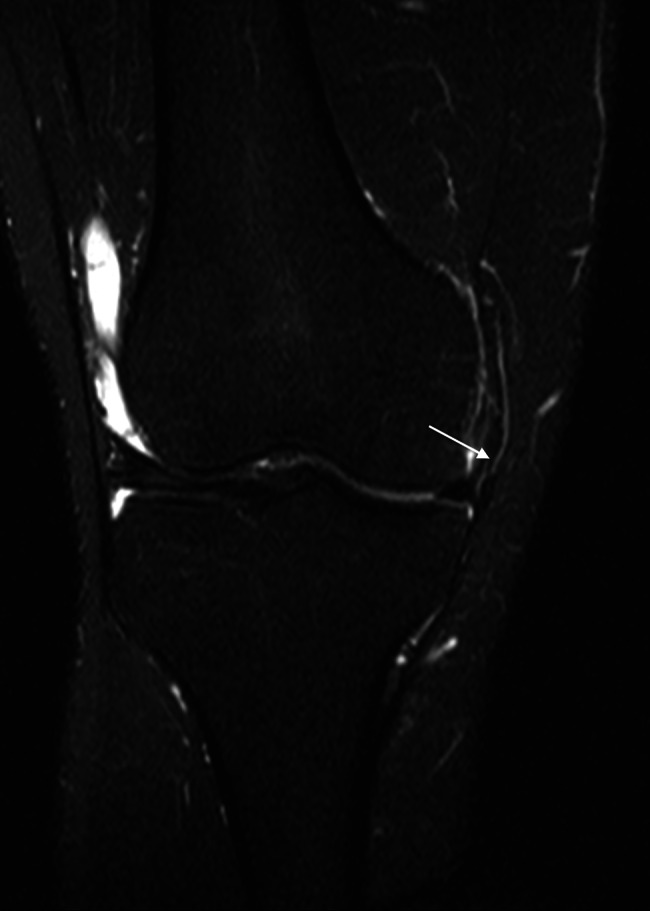
AOL in coronal view presented as a thin, mostly vertically oriented structure, whose femoral origin could not be separated from the sMCL.

However, due to the proximity between these ligaments, their separate origins could not be undoubtedly individualized in MRI. The ligament can be followed running distally, anterior, and inferiorly towards the tibia, maintaining its close relationship with the anterior border of the sMCL until its insertion in the tibia, in the posterior third between the medial collateral ligament (MCL) and the patellar ligament. The tibial insertion is approximately 1 cm distal from the medial tibial plateau. In coronal sequences, the AOL could not be easily identified in the majority of scans, and when possible, it was presented as a thin, mainly vertically oriented structure whose femoral origin could not be separated from the sMCL (Fig. 7). The distal segments of these ligaments could be separated more easily.

In the Anterolateral Ligament initial radiological studies, it was less identified than nowadays. Helito et al., in their first radiological publication, reported that the ALL was best viewed in MRI examinations through sequences acquired in the coronal plane. The ligament was completely characterized only in 33.3% of the cases [14]. The ligament became better visualized with time passage and more systematic studies on it. It is possible to see parts of it in more than 90% of the exams and to see it completely in 71% of the cases [15].

Furthermore, the quantitative results surprisingly showed a significant presence of the anterior oblique ligament in the MRI exams, a structure never noticed before and exceeding our expectations, with a substantial agreement among the observers who, although experienced, only recently learned to visualize the ligament through MRI.

The outcome of this study shows a very relevant finding, considering the results suggest that, although the anterior oblique ligament is a newly discovered structure and, therefore, not routinely evaluated in MRI, it is indeed a consistent structure in the knee, which can frequently be visualized on MRI studies and recognized with a high level of interobserver agreement, once radiologists become aware of its existence and know what to look for. The possibility to visualize the AOL in MRI scans in a relatively large number of exams, as shown in this study, implies that it can reliably be used for further evaluation and studies related to the anterior oblique ligament. 3T scanners are available nowadays, the evaluation with 3T MRI could clarify even better the description of the structure in the future.

### For the use of human tissue samples

- all methods were carried out in accordance with the Institution and the National guidelines and regulations.
- The experimental protocol was approved by the *Santa Casa de São Paulo* ethics committee (CAAE: 34459220.0.0000.5479; number: 4.251.622).
- The informed consent was obtained from all subjects, everybody over 21 years.

## Data Availability

The datasets used and/or analysed during the current study are available from the corresponding author on reasonable request.
